# New Developments in Medicine

**Published:** 1913-04-19

**Authors:** 


### NEW DEVELOPMENTS IN MEDICINE.

### III.—THE PERMANENT BATH.

In England the permanent bath has been generally recognised only in one group of cases—namely, in patients subjected to profound shock by burns, or by diarrhœa in the case of children. But its usefulness in many other conditions has not yet been realised in English hospitals as it is realised in Continental institutions. With us the method of treatment by immersing the patient for a prolonged time in a tepid bath is utilised in children's wards in cases of burns and extensive scalds, and in cases of loss of moisture due to gastro-enteritis associated with tetany. These are practically the only two indications given in most text-books for the use of the permanent bath, and in no case are full directions given how the method is to be used. This obviously shows that the authors have had no practical experience in using the method, and a preliminary inquiry among the staff of certain hospitals has borne out this conclusion. The average nurse or sister does not know how the permanent bath is to be used; she has never seen it used; and she is for the most part unacquainted with the fact that it is used. In a few rare cases where it has been tried, our experience has been that it has been so badly arranged, owing to the want of familiarity with the technique on the part of the staff and nurses, that the patient has not received much benefit from it. In one or two cases actual damage has been done. The method, indeed, cannot rightly be stated to be a new method in medicine, for the permanent bath is a very old institution. Aretæus immersed his patients in brine baths for several days, and Trotula used the permanent bath at Salern in cases of lithiasis, apparently with excellent results to the general health of the patients. But the indications for the use of the bath have recently been laid down on what may be considered scientific lines, and in the past they were by no means certain or accurate, since every practitioner who employed the bath had his own fads.

### The Practice of France and Germany.

Let us see how the permanent bath is used in France and Germany, where it is nowadays to be found in every hospital that lays claim to being modern. Professor Dr. Grober, the superintendent of one of the finest and best of the newer German hospitals, in an article dealing with the planning of hospitals, lays stress on the necessity for the provision of a permanent bath in every ward, so placed that the patient in the bath can be readily watched by the ward nurse. In the general hospital the bath is arranged either in the ward itself—in which case it is regarded as a bed and placed so that it receives the requisite amount of air and direct sunlight—or in a special room opening from the ward, and not, generally, so situated that it is part of the lavatory annexe. These baths are specially useful in the surgical block and in the department for skin diseases. Especially useful are the baths in cases of restless patients. This usefulness is responsible for their present extensive employment in asylums and mental wards. The advantage of the permanent bath is that it gives all the benefits of the best bed treatment. As Professor Weygand remarks: "It brings tranquillity, removes as much as possible all sources of irritation, such as the friction of the bedclothes, stimulates the circulation, improves the appetite, and effectually prevents uncleanliness and bedsores. It is really wonderful to see the rapidity with which large bedsores which have eaten into deep-seated parts are cured in a few days by the bath treatment; even trophic sores in patients who are quite paralysed, as in cases of multiple sclerosis and tumours of the cord, are sensibly improved by the method." There can be no question that the permanent bath is a very desirable adjunct to the ordinary apparatus which the hospital surgeon has at his disposal in cases of burns; there can be equally little doubt that it is as useful on the medical side in a variety of cases which are now treated by old-fashioned methods without much improvement.

### The Type of Bath.

The baths, or bath, as the case may be—for in general, one permanent bath is sufficient for even a large ward—must be carefully considered. Plain porcelain baths are the best, and it is needless to say they must have carefully rounded edges and corners and no sharp points on which a patient can injure himself. The question of taps and tubes presents various difficulties. The best method is, perhaps, to have no taps within the patient's reach at all, but to have the water supply under the control of the nurse some distance away from the bath itself; this is certainly the best method in case of mental hospitals. Proper means must be available to keep the temperature of the bath even and to secure a uniform, steady flow of water without the creation of currents; the only way in which this ideal can be secured is by providing one of the newer patent mixing apparatus—that of Schafstedt is generally used in German mental hospitals—in which special mechanisms secure the evenness of the flow. With these are usually arranged automatic visual indicators which show the variations in temperature on the instant they occur. The complete apparatus acts automatically, is on the whole simple in construction and unlikely to give rise to much trouble, and considerably diminishes the inconveniences and dangers of the permanent bath.

That there are inconveniences and dangers everyone must admit. Patients in these baths need constant supervision and must be carefully watched. The change from one temperature to another—even so slight a rise or fall as one degree—may considerably influence the vasomotor system of the patient in the bath. Patients with respiratory troubles must be most carefully watched; in anæmic subjects the bath often seems to do more harm than good. Above all, the greatest care must be exercised when there is any myocardial degeneration. Patients who are melancholic, with a tendency towards committing suicide, will find ample means to carry out their intentions in the permanent bath unless they are strictly watched. In a few cases skin rashes have been observed as the result of using the permanent bath; in mental hospitals where the bath is much used it is said that the bath *personnel* suffers in health from constant occupation in the bath room, but this danger appears to us to be much over-estimated, since there is no reason why a nurse in attendance on a patient in the permanent bath should suffer in health more than one who nurses any other patient in the ward.

There are a few obvious hints with regard to the technique which may here be given. A patient in the permanent bath need not be naked; in the case of women especially it is advisable to make the patient wear a thin shirt or bathing-dress. The provision of a blanket or sheet on which the patient can lie in the water is sometimes very useful. Special attention must be paid to the support of the head. This is best done by a broad rubber or elastic head-rest, which does not exercise any pressure on the veins of the neck. A cover to the bath is quite unnecessary and even dangerous, nor must the patient's head in any circumstances be covered. Once the technique of the permanent bath is well understood, and the usefulness of this new method of treatment is fully realised, it is only a question of time before it is introduced into all hospitals. The benefits to be derived from it in certain cases are so manifest that it is a wonder they have not appealed to the medical staffs of hospitals before, since everyone who has visited a modern Continental hospital must have seen how useful the *Dauerbad* can be made in certain cases.

